# Role of CD8 Regulatory T Cells versus Tc1 and Tc17 Cells in the Development of Human Graft-versus-Host Disease

**DOI:** 10.1155/2017/1236219

**Published:** 2017-01-09

**Authors:** Adriana Gutiérrez-Hoya, Rubén López-Santiago, Jorge Vela-Ojeda, Laura Montiel-Cervantes, Octavio Rodríguez-Cortés, Víctor Rosales-García, Vladimir Paredes-Cervantes, Raúl Flores-Mejía, Daniela Sandoval-Borrego, Martha Moreno-Lafont

**Affiliations:** ^1^Departamento de Inmunología, Escuela Nacional de Ciencias Biológicas, Instituto Politécnico Nacional, Prolongación de Carpio y Plan de Ayala S/N, Colonia Santo Tomás, Miguel Hidalgo, 11340 Ciudad de México, Mexico; ^2^Cátedras CONACyT, CONACyT, Avenida Insurgentes Sur 1582, Benito Juárez, Crédito Constructor, 03940 Ciudad de México, Mexico; ^3^Departamento de Diferenciación Celular y Cáncer, FES Zaragoza, Universidad Nacional Autónoma de México, Batalla 5 de mayo S/N Esq. Fuerte de Loreto, Iztapalapa, 04650 Ciudad de México, Mexico; ^4^Unidad Médica de Alta Especialidad, Centro Médico Nacional La Raza, Instituto Mexicano del Seguro Social, Seris y Zaachila S/N, Col. La Raza, Ciudad de México, Mexico; ^5^Departamento de Inmunología Médica, Escuela Superior de Medicina, Instituto Politécnico Nacional, Calle Plan de San Luis y Díaz Mirón S/N, Casco de Santo Tomas, Miguel Hidalgo, 11340 Ciudad de México, Mexico; ^6^Laboratorios Nacionales de Servicios Experimentales, Centro de Investigación y de Estudios Avanzados, Instituto Politécnico Nacional, Av. IPN 2508, Colonia San Pedro Zacatenco, 07360 Ciudad de México, Mexico; ^7^Hospital General, Centro Médico Nacional La Raza, Instituto Mexicano del Seguro Social, Apartado Postal 14-878, 07001 Ciudad de México, Mexico

## Abstract

CD8^+^ T cells that secrete proinflammatory cytokines play a central role in exacerbation of inflammation; however, a new subpopulation of CD8 regulatory T cells has recently been characterized. This study analyzes the prominent role of these different subpopulations in the development of graft-versus-host disease (GVHD). Samples from 8 healthy donors mobilized with Filgrastim® (G-CSF) and 18 patients who underwent allogeneic hematopoietic stem cell transplantation (HSCT) were evaluated by flow cytometry. Mobilization induced an increase in Tc1 (*p* < 0.01), Th1 (*p* < 0.001), Tc17 (*p* < 0.05), and CD8^+^IL-10^+^ cells (*p* < 0.05), showing that G-CSF induces both pro- and anti-inflammatory profiles. Donor-patient correlation revealed a trend (*p* = 0.06) toward the development of GVHD in patients who receive a high percentage of Tc1 cells. Patients with acute GVHD (aGVHD), either active or controlled, and patients without GVHD were evaluated; patients with active aGVHD had a higher percentage of Tc1 (*p* < 0.01) and Tc17 (*p* < 0.05) cells, as opposed to patients without GVHD in whom a higher percentage of CD8 Treg cells (*p* < 0.01) was found. These findings indicate that the increase in Tc1 and Tc17 cells is associated with GVHD development, while regulatory CD8 T cells might have a protective role in this disease. These tests can be used to monitor and control GVHD.

## 1. Introduction

Graft-versus-host disease (GVHD) is one of the major causes of mortality after allogeneic hematopoietic stem cell transplantation (HSCT); it is induced by the inflammatory immune response of donor cells against host tissues recognized as foreign. It is usually referred to as acute GVHD (aGVHD) when damage appears within the first 100 days after allogeneic HSCT and the main organs involved are the skin, liver, and gastrointestinal tract. The development of this disease depends on diverse immunological characteristics of the patient and donor at the time of infusion [1–3].

A central aspect and a subject of evaluation in GVHD development is the role of cytokines. In this context, GVHD has been extensively associated with Th1-related cytokines (IFN*γ*, IL-2, and IL-12) [4, 5] although these are not the only cytokines involved in inflammation. Recently, Th17-related cytokines (IL-17A and IL-17F) have been said to be prominent in solid organ rejection in murine models [6–9] and while their presence is not required for GVHD development, they contribute to exacerbation of this disease [8].

As a counterpart to inflammation and as part of homeostasis, a beneficial process known as immune regulation takes place. Research on this subject has focused on the study of regulatory T cells (Treg), in particular those that express the CD4^+^CD25^high^FoxP3^+^ phenotype, which are able to control immune responses to alloantigens and are therefore potential targets for establishing tolerance in transplantation. This Treg subpopulation has been studied the most; however, other groups of cells with regulatory functions have been described, for example, the subsets CD8^+^, *γδ* T, NK, and NKT. This is the reason why many studies are now focusing on them in order to promote an immune tolerance status via the adoptive transfer of these cells [10, 11]. Within this context are CD8^+^ Treg, initially described by Gershon and Kondo (1970) [12], the study of which was abandoned due to the lack of markers to characterize them and has recently been taken up in clinical studies that have established their role in diverse diseases such as experimental autoimmune encephalitis [13–15], colorectal cancer [16, 17], multiple myeloma [18, 19], multiple sclerosis [20], and ovarian carcinoma [21, 22]. These findings demonstrate the prominent immunosuppressive role of CD8^+^ cells in control of autoimmunities and evasion of the immune response. These antecedents, together with CD8^+^ Treg generation through continuous stimulation of the antigen [23] and involvement of these cells in GVHD control in murine models [24], denote the importance of the regulatory functions carried out by CD8^+^ cells. Nevertheless, there are no studies on the role of CD8^+^ Treg in GVHD development in humans, and findings regarding proinflammatory Tc17 cells are few and controversial.

The present study was designed to determine the utility of Tc1 and Tc17 cells, as opposed to CD8^+^ Treg, as predictors of GVHD development and severity.

## 2. Materials and Methods

### 2.1. Patients and Donors

Eighteen human leukocyte antigen- (HLA-) identical sibling donors and their recipients with different hematooncologic disorders were studied. Half of the patients developed GVHD (55.5%). All individuals complied with the requirements to be included in the Stem Cell Transplantation Program at Centro Médico Nacional La Raza (IMSS-Mexico) and signed an informed consent before entering the study. The Hospital Ethical Committee approved the study, which was conducted according to the principles of the Declaration of Helsinki.

Blood samples were obtained on months 1, 2, 6, 9, and 12 after transplantations. All patients were clinically evaluated on a monthly basis for GVHD development.

Granulocyte-colony stimulating factor (G-CSF) (Filgrastim, Amgen-Roche, Thousand Oaks, CA) was subcutaneously administered to donors in daily doses of 16 *μ*g/kg for five days. Apheresis was performed on day 5 of G-CSF administration using a Cobe Spectra device. Patient characteristics are shown in Table 1. Patients with cytomegalovirus (CMV) infection were excluded from the study.

### 2.2. Determination of Lymphocyte Subpopulations and Cytokines by Flow Cytometry

**Figure 1:**
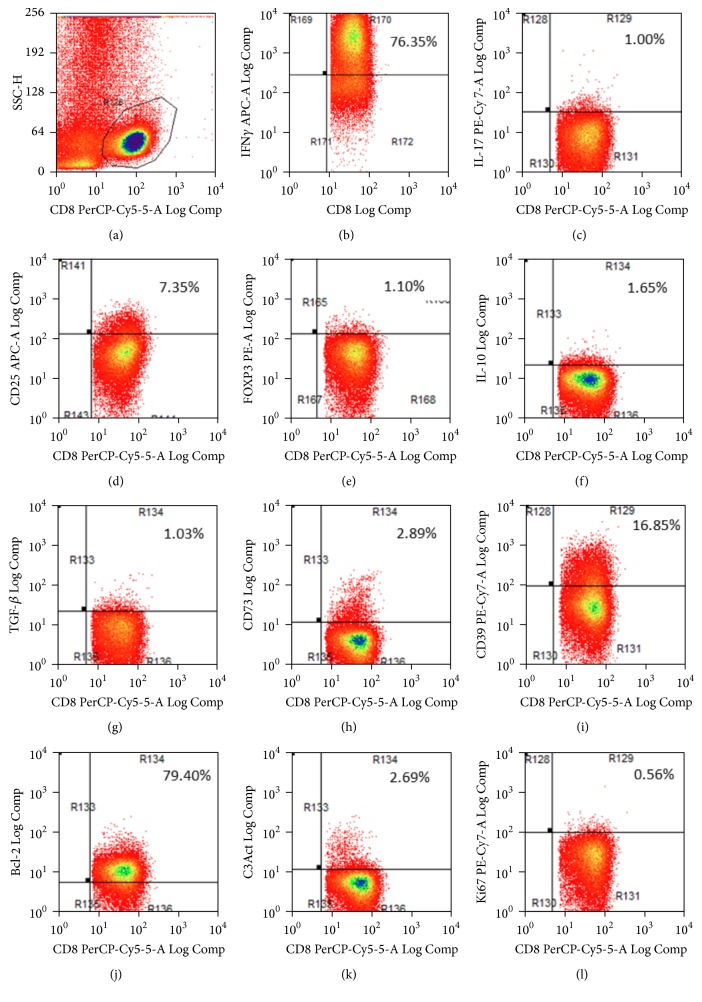
Characterization of CD8^+^ cells by flow cytometry. (a) CD8^+^ gating. (b)–(l) Determination of (b) IFN*γ*; (c) IL-17; (d) CD25; (e) FoxP3; (f) IL-10; (g) TGF*β*; (h) CD73; (i) CD39; (j) Bcl-2; (k) active caspase-3; and (l) Ki-67.

Peripheral blood (PB) samples were obtained, stained for multiparametric flow cytometry, and fractionated in 4 Eppendorf tubes (A, B, C, and D) containing 500 *μ*L of blood each. The sample in tube A was unstimulated; tube B was stimulated with brefeldin A plus monensin; tube C was stimulated with 40 ng PMA plus 1 *μ*g ionomycin; tube D was stimulated with 40 ng of PMA and 1 *μ*g ionomycin plus brefeldin A and monensin. Samples were incubated at 37°C in a 5% CO_2_ atmosphere for 6 h. The following antibodies were used for cell surface staining: mouse anti-human CD4 PE-TexRed or CD4 PerCP, CD8 PE-TexRed, CD8-PerCP, CD25-APC (BioLegend, San Diego, CA), CD8-FITC, CD8-PE, or CD3-PerCP (BD, San Jose, CA). Cells were incubated for 20 min at 4°C in staining buffer (PBS, 0.5% BSA, and 0.01% sodium azide). The cell suspensions were then washed, lysed, and permeabilized for intracellular staining with anti-human-TGF*β*-FITC, Bcl-2-FITC, active caspase 3-FITC, Ki67-PE-Cy7, Ki67-PerCP, IFN*γ*-APC, IL17-FITC, and IL17-PE-Cy7 (BioLegend). The tubes were incubated for 20 min at 4°C in the dark. Finally, the cells were washed, fixed with 1% paraformaldehyde, and analyzed in a flow cytometer (FACSAria III, BD) (Figure 1).

### 2.3. Statistical Analysis

Differences between patients with and without GVHD were analyzed with a Kruskal-Wallis test followed by Dunn's multiple comparisons test, while differences between donors before and after administration of G-CSF were evaluated with a Mann–Whitney* U* test (GraphPad Prism v5.0). Kaplan-Meier curves were used to evaluate survival. *p* values <0.05 (*∗*), <0.01 (*∗∗*), and <0.001 (*∗∗∗*) were considered significant.

## 3. Results and Discussion

### 3.1. G-CSF-Based Mobilization Induces Proinflammatory Cytokines but Not Cell Death

To detect whether mobilization with G-CSF induces proinflammatory cytokines and/or cell death, the expression of IFN*γ* and IL-17 as inflammation markers was determined, while Bcl-2 and active caspase-3 were used to evaluate the viability of CD4^+^ and CD8^+^ cells. Determinations were performed on two groups of healthy individuals: a control group (*n* = 6) and a group of G-CSF-mobilized donors (*n* = 8). Results show that mobilization induces an increase in the percentage of Th1 (*p* ≤ 0.001), Tc1 (*p* ≤ 0.01), and Tc17 (*p* ≤ 0.05) cells. There was a trend to increase in the percentage of Th17 cells (*p* = 0.08). It is worth noting that this increase was higher in type 1 cells.

**Figure 2:**
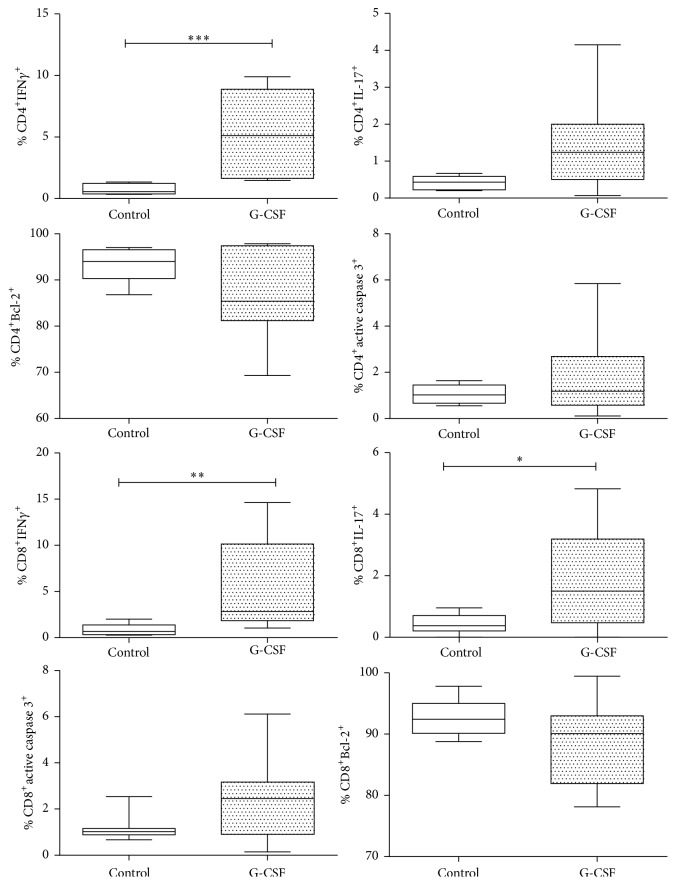
Cell viability and proinflammatory cytokines in healthy donors mobilized with G-CSF. Determination of IFN*γ*, IL-17, Bcl-2, and active caspase-3 in peripheral blood CD4^+^ and CD8^+^ cells from a control group (*n* = 6) and a group of G-CSF-mobilized donors (*n* = 8). Box plots show population distribution and whiskers denote one standard deviation. A significant increase in the number of CD4^+^IFN*γ*^+^ (Th1), CD8^+^IFN*γ*^+^ (Tc1), and CD8^+^IL-17^+^ (Tc17) cells is observed; this increase is not significant in CD4^+^IL-17^+^ (Th17) cells, but a marked tendency is shown (*p* = 0.06). ^*∗*^*p* < 0.05; ^*∗∗*^*p* < 0.01; ^*∗∗∗*^*p* < 0.001.

To assess whether mobilization affects cell viability, active caspase-3 was determined as an apoptotic marker and Bcl-2 as an antiapoptotic marker. Results show that mobilization does not induce death on both CD4^+^ and CD8^+^ T cells (Figure 2).

GVHD is a process characterized by exacerbation of the inflammatory immune response and absence of immune regulation. In this context, CD8^+^ cells play a fundamental role due to their rapid reconstitution after allogenic HSCT, as well as the fact that they constitute the major population in the transplant recipients. For years these cells have been considered preeminently cytotoxic, having the capacity to secrete proinflammatory cytokines; however, recent data in murine models indicate the existence of a subpopulation of CD8^+^ regulatory T cells with functional characteristics that may contribute to control of GVHD [25–27].

There is an evident need to evaluate the effect of mobilization on CD8^+^ cells in donors and its correlation with GVHD development in patients, since prior reports indicate that G-CSF is able to induce diverse immunological profiles; for example, some investigators propose that mobilization promotes Th2 response while mitigating Th1 response [28, 29], while others report that mobilization in mice induces proinflammatory type 1 and type 17 cells [30]. Zhao et al., 2011, conclude that, in in vivo human samples, G-CSF inhibits production of Th17 cells in bone marrow (BM) and PB grafts [31]. In our study a significant increase was observed in the percentage of proinflammatory Th1: CD4^+^IFN*γ*^+^ (*p* < 0.001), Tc1: CD8^+^IFN*γ*^+^ (*p* < 0.01), and Tc17: CD8^+^IL-17^+^ (*p* < 0.05) cells and this increase was not significant in Th17 cells (*p* = 0.08). It is important to note that this increase was higher in Th1 and Tc1 cells (Figure 2). These results indicate that mobilization with G-CSF induces proinflammatory cell types, and that this may affect patients who receive such cells.

### 3.2. G-CSF-Based Mobilization Induces an Increase in the Percentage of Proliferating and IL-10-Positive CD8^+^ Cells

**Figure 3:**
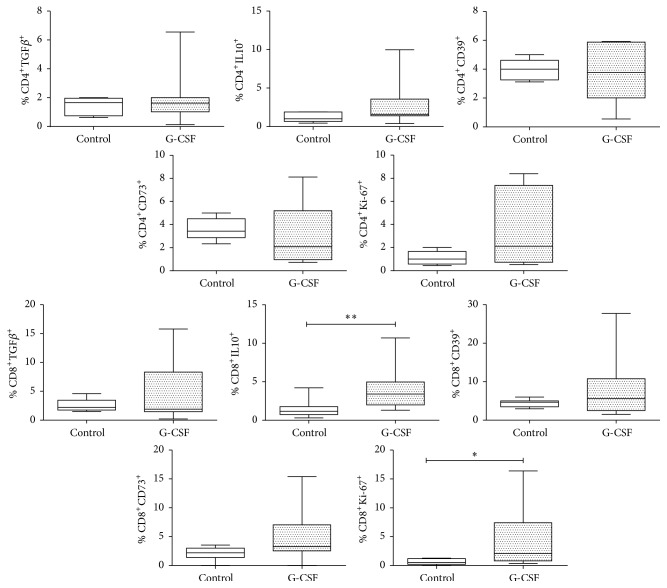
Cell viability and anti-inflammatory molecules in healthy donors mobilized with G-CSF. Determination of TGF*β*, IL-10, CD39, CD73, and Ki-67 in peripheral blood CD4^+^and CD8^+^ cells from a control group (*n* = 6) and a group of G-CSF-mobilized donors (*n* = 8). Box plots show population distribution and whiskers denote one standard deviation. A significant increase is observed in the number of CD8^+^IL-10^+^ and CD8+Ki67^+^ cells; the change in CD8^+^CD73^+^ cells is not significant, but a marked tendency to increase is shown (*p* = 0.06); no changes or tendencies are seen in marker expression in CD4^+^ cells. ^*∗*^*p* < 0.05; ^*∗∗*^*p* < 0.01.

To find whether mobilization with G-CSF induces anti-inflammatory molecules and/or cell proliferation, the expression of the regulatory molecules TGF*β*, IL-10, CD39, and CD73 was evaluated, and Ki-67 was determined in CD4^+^ and CD8^+^ cells from the control group (*n* = 6) and the group of mobilized donors (*n* = 8). Results indicate that mobilization induces an increase in the number of CD8^+^Ki-67^+^ (*p* ≤ 0.05) and CD8^+^IL-10^+^ (*p* ≤ 0.01) cells and that while the increase in CD8^+^CD73^+^ cells is not significant, a trend was observed (*p* = 0.06). However, no differences or tendencies were observed in regard to CD4^+^ cells (Figure 3).

A significant increase was also observed in the percentage of CD8^+^IL-10^+^ cells (*p* < 0.01), indicating that a regulatory phenotype was induced (Figure 3). This is consistent with previous reports indicating that G-CSF-induces an increase in the number of IL-10-positive cells, as well as other reports in which the number of CD4^+^ Treg and some of their regulatory molecules also increased [32, 33]. It is worth noting that this increase was not induced in CD8^+^TGF*β*^+^ cells, a cytokine being widely recognized for its regulatory role [34]. Other molecules evaluated in the present study that are involved in immune regulation were CD39 and CD73 [35–37], but no significant differences were found. However, a tendency to increase was found in the percentage of CD8^+^CD73^+^ cells (*p* = 0.06) (Figure 3), which may indicate a Treg population since CD73 is a molecule which has been extensively described as a marker of CD4 Treg and is known to carry out regulation via depletion of ATP in the medium in order to inhibit activation [35]. This piece of information is important since there are no reports of the effect induced by G-CSF on this molecule, and this indicates induction of a regulatory phenotype in response to mobilization.

### 3.3. Donor-Patient Correlation

**Figure 4:**
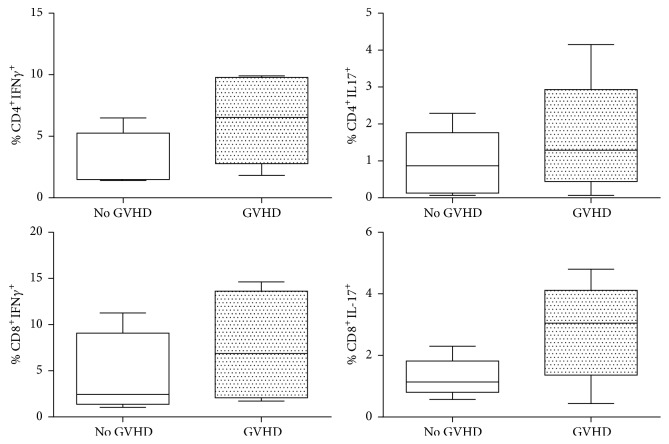
Correlation of patients with GVHD and without GVHD and percentage of type 1 and type 17 cells in the corresponding donor. No significant differences are seen, but a correlation (*p* = 0.06) exists, which indicates that patients who develop GVHD are patients who received a higher percentage of Th1 cells (CD4^+^IFN*γ*^+^).

Having seen that mobilization with G-CSF induces an increase in type 1 and type 17 cells, the next question was to determine whether the activation status of these cells influenced GVHD development in the patient. Two groups of patients, with and without GVHD, were tested for correlations with donor response to mobilization. The results obtained were not significant, but a trend (*p* = 0.06) was observed in patients who receive a higher percentage of Th1 cells that are more susceptible to develop GVHD (Figure 4).

Knowing that G-CSF may polarize into proinflammatory and regulatory phenotypes and, even more notably, that type 1 and type 17 cells are induced, it was decided to examine the donor-patient correlation in order to determine if a link exists between the activation status of cells infused in the patient and GVHD development. Donor cell response was therefore tested for correlations with patients who developed aGVHD and those who did not. No significant differences were observed, but a tendency was noticed in the case of CD8^+^IFN*γ*^+^ cells (*p* = 0.06), suggesting that donors who respond to mobilization with a higher percentage of these cells are the donors of patients who develop aGVHD (Figure 4).

The following step was to follow up patients after allogeneic HSCT and to evaluate GVHD development. Three groups of patients were evaluated: patients with active GVHD, without GVHD, and with controlled GVHD. A wide panel of biomarkers was studied on CD8^+^ cells of these patients, since this is one of the earlier cell populations to be reconstituted after allogeneic HSCT [38].

### 3.4. CD8 Treg versus Tc1 and Tc17 Cells in Development and Severity of GVHD

**Figure 5:**
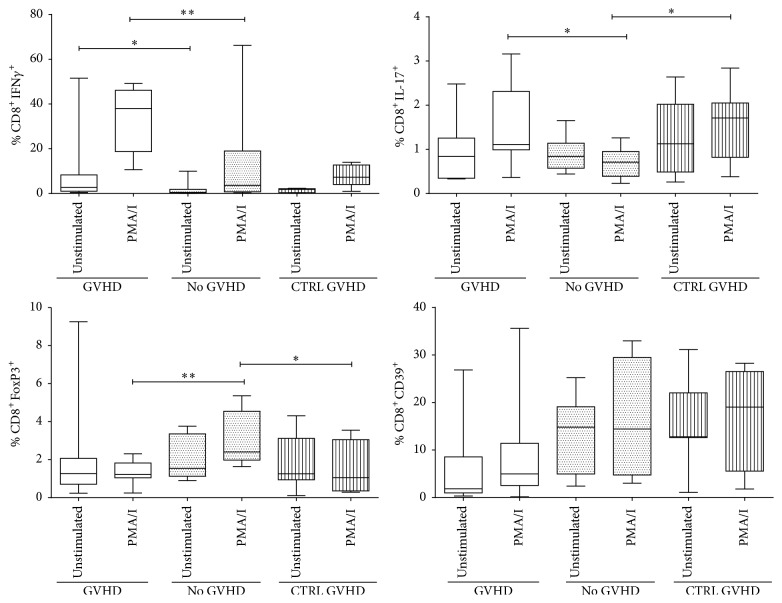
Determination of Tc1, Tc17, and CD8^+^ Treg in patients without GVHD, with active GVHD, and with controlled GVHD. Patients with active GVHD show an increase in Tc1 and Tc17 cells, while patients without GVHD display an increase in CD8^+^FoxP3^+^ cells compared to patients with active or controlled GVHD. ^*∗*^*p* < 0.05; ^*∗∗*^*p* < 0.01.

To evaluate the role of the cells that express proinflammatory or regulatory phenotypes in GVHD development, regulation-related markers (TGF*β*, IL-10, CD39, CD73, and FoxP3) and inflammation markers (IFN*γ*, IL-17, and CD25) were evaluated in patients without GVHD, as well as patients with grade III or IV GVHD during the active phase of the disease and once symptoms were controlled (controlled GVHD). Eighteen patients entered the study group; however, patients with CMV, aspergillus, and/or herpes zoster virus (HZV) infection were excluded. A higher percentage of unstimulated and stimulated Tc1 cells was observed in patients with active GVHD (*p* ≤ 0.01 and *p* ≤ 0.05, resp.), compared to those without GVHD (Figure 5). On the other hand, Tc17 cells increased in patients with active and controlled GVHD, compared to patients without GVHD, but this increase was evidenced only after polyclonal activation with PMA and ionomycin. These results suggest that Tc1 and Tc17 cells play a prominent role in GVHD. A further aspect evaluated was the involvement of CD8 Treg in GVHD development. To this end, cells described as regulatory in other pathologies, that is, CD8^+^CD39^+^, CD8^+^TGF*β*^+^, CD8^+^IL-10^+^, CD8^+^CD25^+^, CD8^+^CD73^+^, and CD8^+^FoxP3^+^ cells, were evaluated. No significant differences or tendencies were observed in the first four of these subsets. On the other hand, the number of CD8^+^FoxP3^+^ cells increased in patients without GVHD compared to patients with active or controlled GVHD (*p* ≤ 0.01 and *p* ≤ 0.05, resp.). Interestingly, other cells involved in regulation (i.e., CD8^+^CD39^+^) showed a tendency to increase in patients without GVHD, compared to patients with either active or controlled GVHD (Figure 5).

To evaluate induction of proinflammatory cytokines, IFN*γ* and IL-17 were determined. An increase in the percentage of Tc1 cells was found in patients with active GVHD compared to patients without GVHD, these differences occurred at basal levels and in response to polyclonal stimulation (*p* < 0.01 and *p* < 0.05, resp.), their impact being greater at basal levels (Figure 5). This is consistent with diverse reports in human and murine models in which an increase in Th1 cells in PB and higher serum levels of IFN*γ* are evidenced in patients with GVHD [4, 5, 39, 40]. The role of Tc17 cells was also evaluated since these cells have recently been proposed to be implicated in inflammation and were linked to GVHD development and severity; however, results in human and murine models are contradictory [41–43]. In the present study, an increase in the percentage of Tc17 cells was found in patients with active GVHD compared to patients without GVHD (*p* < 0.05). This is in agreement with data reported in murine models [8]; however, it is important to note that this increase was also found in patients with controlled GVHD compared to those without GVHD (*p* < 0.05) and that in both cases the increase was evidenced only after polyclonal stimulation; this was unexpected. However, the fact that it was seen only after polyclonal activation suggests that once the disease is controlled, these cells do not receive damage signals that induce them to migrate to target organs and therefore are consequently found in PB. It should be made clear that these cells are preferentially found in the mucosae. Moreover, the group of Zhao et al. [44] found that the differentiation of cytokine-producing Tc1 and Tc17 cells may be the key step in the initiation of GVHD, whereas Th1 and Th17 cells are considered to be a pathophysiological factor leading to the continuous aggravation of GVHD this antecedent support our findings.

The following aspect to be evaluated was regulatory molecules expression. A wide range of regulation-related markers was determined, revealing the prominent role of CD8 Treg. A decrease in the percentage of these cells occurred in patients with active GVHD compared to those without GVHD (*p* < 0.01); a similar finding was observed in patients with controlled GVHD but with a lower level of significance (*p* < 0.05), probably indicating reconstitution of this cell subpopulation (Figure 5). It is worth noting that there are no data on the role of these cells in GVHD development in humans; the only prior report is in murine models by Beres et al., 2012 [25], reporting this subpopulation as a possible regulator of GVHD development. On the other hand, Zheng et al. [45] found in a model of GVHD in humanized mice how the allogenic-specific CD8 Treg controlled the development of GVHD in an allospecific manner by reducing alloreactive T cell proliferation, decreasing inflammatory cytokines as IFN*γ*, IL-17, TNF*α*, IL-6, IL-2, and IL-1*β* as well as chemokine secretion through a CTLA-4 dependent mechanism. Other reports on the role of CD8^+^ Treg in malignant tumors associate this subset of Treg cells with poor prognosis as well as with the severity in multiple sclerosis [16, 21, 22, 46]. Other regulatory molecules were also evaluated such as TGF*β*, IL-10, and CD73 but no significant differences were observed (data not shown), while a tendency to decrease was seen in the percentage of CD8^+^CD39^+^ cells in patients with active GVHD, compared to those without GVHD. This may imply that this subset of T cells plays a prominent role in GVHD development. As far as today, the only reports are available in murine models and show that blocking CD73+ cells potentiates GVHD development [47], while another study shows that Treg from CD73 KO mice are less effective than WT Treg in suppression of GVHD [48]. However, there is no information in regard to the role of CD39 in GVHD development, and it was therefore decided to analyze these molecules. No significant differences were found, although a tendency to increase is observed in the percentage of CD8^+^CD39^+^ cells in patients with active GVHD.

Despite the significant progress that has been made in understanding the role of CD8 Treg cells in GVHD development, a number of questions remain. First of all, we do not know if the CD8+FoxP3+ cells are able to suppress the in vivo and in vitro immune responses. Secondly, the major problem in the study of CD8+ Treg is the lack of surface or intracellular specific markers that unequivocally define these cells as suppressors. FoxP3 expression is not exclusive to regulatory T cells and its expression is unstable. For that reason, new markers for isolation and testing Treg suppression function have to be found. Additional studies are required to determine whether the CD8 Treg migrate to GVHD target organs and determine if they are functional.

Although our results suggest a role for functional regulatory CD8 T cell population in the control of GVHD, the mechanisms remain unknown, since apparently the regulation may be independent of the classical molecules, that is, TGF*β* or IL-10. A study on the detailed molecular response of the CD8 Treg in similar controlled groups of donors must be done to well understand the mechanisms of regulation of the GVHD in allogenic transplants.

## 4. Conclusions

**Figure 6:**
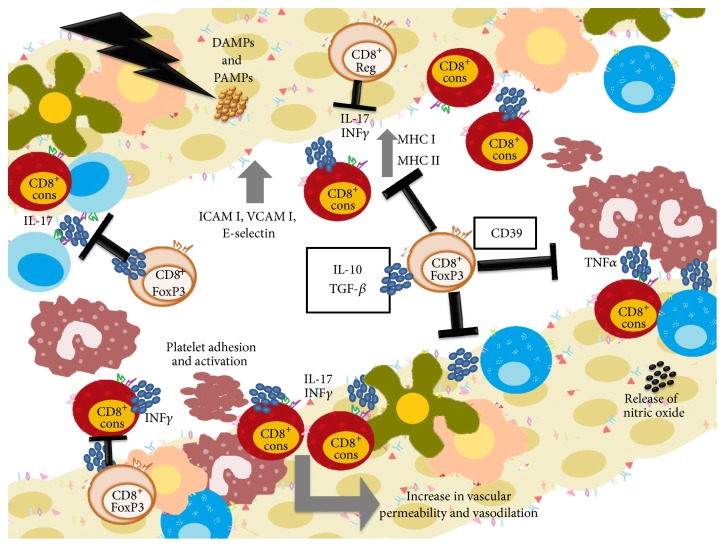
Peripheral regulatory mechanism that controls the development of GVHD. The development of GVHD is characterized by activation of T donor cells. Donor T cell activation results mainly in IFN*γ* and IL-17 production; the IFN*γ* promotes the increase of MHC, adhesion molecules, nitric oxide release, and vasodilation and increased permeability. This results in further increases in antigen presentation and activation and expansion of cytotoxic CD8^+^ and CD4^+^ T cells; these cells migrate to the target organs, where they mediate tissue injury that leads to multiorgan failure. Nevertheless, there are subpopulations that can intervene at different stages of GVHD and control it; the subpopulation named CD8^+^ Treg can intervene and block activation and expansion of effector cells, through IL-10 and TGF*β* production and expression of CD39; these cells reduce the inflammatory state.

The recently described CD8 Treg subpopulation was identified by our working group for the first time in patients undergoing allogeneic hematopoietic transplantation, together with its fundamental role in GVHD control in humans. The results obtained show the important role of proinflammatory Tc1 and Tc17 cells in GVHD severity and development. A further finding is the polarizing effect of G-CSF in donors, inducing proinflammatory Th1, Tc1, and Tc17 cells and anti-inflammatory CD8^+^IL-10^+^ cells, which may affect early GVHD development. Therefore, the use of Tc1 and Tc17 cells as negative predictive indicators for development and severity of GVHD and CD8 Treg as positive predictive indicators for control of GVHD is proposed, as well as monitoring of these cells in mobilized samples (Figure 6).

## Figures and Tables

**Table 1:** Patient characteristics.

Number of patient	Sex	Age (years)	Disease	Degree of GVHD
01	F	49	AML M2	I
02	F	32	NHL IIIB	I
03	F	19	MDS	IV
04	M	55	MDS	III
05	M	56	MDS	CMV
06	M	44	AA	0
07	M	35	AA	I
08	M	18	NHL	HSV
09	F	26	AA	I
10	M	39	AML M5	III
11	M	28	ALL L2	III
12	F	18	AML M5	IV
13	F	32	AA	0
14	F	37	AA	Multiple bacterial infections
15	M	44	AML M2	III
16	F	46	Biphenotypic leukemia	0
17	M	29	AA	0
18	M	44	AA	III

AA: aplastic anemia; AML: acute myeloid leukemia; MDS: hypoplastic myelodysplastic syndrome; NHL: non-Hodgkin lymphoma; ALL: acute lymphoblastic leukemia; GVHD: graft-versus-host disease; HSV: herpes simplex virus; CMV: cytomegalovirus.
